# PD GENEration: An International Parkinson’s Disease Genetic Research Study

**DOI:** 10.64898/2026.05.20.26353696

**Published:** 2026-05-22

**Authors:** Kamalini Ghosh Galvelis, Allison A. Dilliott, Megan Dini, Rebeca De León, Max Thom, Ignacio Azcarate, Nicola Bothwick, Lark Caboy, Anny Coral-Zambrano, Kirby Doshier, Megan Finke, Melissa Nicewaner, Sarah Osborne, Joshua Ruffner, Addison Yake, Adolfo Diaz, Tatiana Foroud, Anne Hall, Laura Heathers, Sarah Woody Lawrence, Karen Marder, Ignacio Mata, Niccolò E. Mencacci, Anna Naito, Martha Nance, John Poma, Ruth B. Schneider, Michael A. Schwarzschild, Tanya Simuni, Jennifer Verbrugge, Anne-Marie Wills, Yun Lu, Harry Gao, Ben Casavant, Cornelis Blauwendraat, Andrew B. Singleton, James C. Beck, Roy N. Alcalay, The Parkinson’s Foundation PD GENEration Study

## Abstract

**Background:**

PD GENEration ( NCT04057794 , NCT04994015 ), sponsored by the Parkinson’s Foundation in partnership with Aligning Science Across Parkinson’s (ASAP) through the Global Parkinson’s Genetics Program (GP2), is an international, observational, clinical research study that offers genetic testing and counseling to people living with Parkinson’s disease (PwP) at no cost. PD GENEration has aimed to empower PwP and their clinicians with knowledge of their genetic status, to accelerate recruitment into precision medicine trials, and to advance research through data sharing. Since its launch in 2019, the study has expanded to enroll over 32,000 PwP (as of March 31, 2026), from 10 countries across North, Central, and South America, the Caribbean, and Israel.

**Methods:**

Over the course of 6 years, PD GENEration has evolved to accommodate the growing scientific and research needs of the Parkinson’s community while also increasing the ability to return genetic test results to PwP at a greater scale. Participants with a diagnosis of Parkinson’s disease (PD) may enroll in-person or virtually where informed consent and blood sample collection can occur. Samples are analyzed at a College of American Pathologists/Clinical Laboratory Improvement Amendments (CAP/CLIA)-certified laboratory using whole genome sequencing, with variants curated for a primary panel of seven PD-associated genes. Results are disclosed during a genetic counseling visit, where further testing is offered for two optional additional gene panels. Those who consent undergo analysis of additional genes, and results are returned during a genetic counseling visit for those that test positive for a variant. In addition to returning genetic results to PwP, a central pillar of the study design has been the open sharing of genomic data to advance discovery in PD research in partnership with ASAP and GP2.

**Discussion:**

PD GENEration applies a flexible framework, allowing for country specific considerations and the integration of multiple site models, evolving based on participant needs and the prioritization of equity and accessibility. We summarize PD GENEration’s implementation and scaling, highlight key accomplishments and lessons learned, and provide guidance for those interested in implementing large-scale clinical genetic testing studies across other diseases and therapeutic domains.

## Full Text Availability

The license terms selected by the author(s) for this preprint version do not permit archiving in PMC. The full text is available from the preprint server.

