# Supplementary material for: PD GENEration: An International Parkinson’s Disease Genetic Research Study
Source: medRxiv. 2026 Aug 28:2026.05.20.26353696. Originally published 2026 May 22. Preprint. [Version 2] doi: 10.64898/2026.05.20.26353696 (PMC13228786; doi:10.64898/2026.05.20.26353696)
Supplement: Supplementary file 2 [file media-2.pdf]

Supplementary Material: PD GENEration Affiliations for Banner Authorship

This supplemental document provides a list of affiliated members of the PD GENEration study under banner authorship for the associated manuscript as per the PD GENEration publication policy (*Rev 05/26*). Affiliations reflect institutional membership at the time of submission.

PDGENE Affiliation	Name	Affiliation(s)
Local Site Investigator	Pinky Agarwal, MD	Evergreen Health, Seattle, WA
Local Site Investigator; National Recruiting Site Investigator	Julian Agin-Liebes, MD	Columbia University, New York, NY
Principal Investigator; Local Site Investigator; Steering Committee	Roy N. Alcalay, MD, MS	Tel Aviv Sourasky Medical Center, Tel Aviv, Israel<br>Columbia University, New York, NY
LATAM Site Investigator	Dr. Sarael Alcauter, PhD	Universidad Autónoma de México, Santiago de Querétaro, QRO, Mexico
Local Site Investigator	Jason Aldred, MD	Inland Northwest Research, Spokane, WA
Local Site Investigator	Irene Andonia Malaty, MD	University of Florida, Gainesville, FL
Local Site Investigator	Joy Antonelle de Marcaida, MD	Hartford HealthCare, Hartford, CT
Genetics Testing Core	Deion Anunciacion	Fulgent Genetics, Temple City, CA
Core Team	Ignacio Azcarate	Parkinson's Foundation, New York, NY
Previous Site and Clinical Data Management Core	Debbie Baker	Clinical Trial Coordination Center (CTCC), Rochester, NY
Local Site Investigator	Matthew Barrett, MD	Virginia Commonwealth University, Richmond, VA
Core Team; Steering Committee; Study Investigator	James C. Beck, PhD	Parkinson's Foundation, New York, NY
Blood Collection Partner	Erwin Berthier, PhD	Tasso Inc, Seattle, WA
Site and Clinical Data Management Core; Steering Committee	Renuka Bhisetti Makhija	Navitas Clinical Research, Rockville, MD
Local Site Investigator	Karen Blindauer, MD	Medical College of Wisconsin, Milwaukee, WI

PDGENE Affiliation	Name	Affiliation(s)
Core Team	Nicola Bothwick	Parkinson's Foundation, New York, NY
Local Site Investigator	Chantale Branson, MD	Morehouse College, Atlanta, GA
Local Site Investigator	Susan Bressman, MD	Mount Sinai Beth Israel Medical Center, New York, NY
Local Site Investigator; National Recruiting Site Investigator	Jeff Bronstein, MD, PhD	University of California Los Angeles (UCLA), Los Angeles, CA
Local Site Investigator	Michiko Bruno, MD	Queen's Health System, Honolulu, HI
Core Team	Lark Caboy	Parkinson's Foundation, New York, NY
Genetics Counseling Core	Valentina Caceres, MS, CGC	Indiana University, Indianapolis, IN
Previous Site and Clinical Data Management Core	Cindy Casaceli	Clinical Trial Coordination Center (CTCC), Rochester, NY
Blood Collection Partner	Ben Casavant, PhD	Tasso Inc, Seattle, WA
LATAM Site Investigator	Pedro Chaná, MD	Corporación Centro de Trastornos del Movimiento, Santiago, Chile
Local Site Investigator	Kelvin Chou, MD	University of Michigan, Ann Arbor, MI
Genetics Counseling Core	Lola Cook, MS, CGC3	Indiana University, Indianapolis, IN
Core Team	Anny Coral Zambrano	Parkinson's Foundation, New York, NY
LATAM Site Investigator	Dr. Mario Cornejo Olivas, MD	Instituto Nacional de Ciencias Neurológicas, Lima, Perú
LATAM Site Investigator	Dr. Rossy Cruz Vicioso, MD	Unión Médica, Clínica Universitaria, Santiago de los Caballeros, Dominican Republic
Local Site Investigator	Nabila Dahodwala, MD	University of Pennsylvania, Philadelphia, PA
Local Site Investigator	Tom Davis, MD	Vanderbilt University, Nashville, TN
Core Team	Rebeca De Leon	Parkinson's Foundation, New York, NY
Local Site Investigator	Marissa Dean, MD	University of Alabama Birmingham, Birmingham, AL

PDGENE Affiliation	Name	Affiliation(s)
Local Site Investigator	Amanda Deligtisch, MD	University of New Mexico, Albuquerque, NM
Local Site Investigator	Rohit Dhall, MD	University of Arkansas, Fayetteville, AR
Core Team	Allison A. Dilliott, PhD	Parkinson's Foundation, New York, NY
Core Team	Megan Dini, MA	Parkinson's Foundation, New York, NY
Local Site Investigator	Elizabeth Disbrow, PhD	Louisiana State University, Baton Rouge, LA
Core Team	Kirby Doshier, PhD	Parkinson's Foundation, New York, NY
Previous Site and Clinical Data Management Core	Ken Eaton	Clinical Trial Coordination Center (CTCC), Rochester, NY
LATAM Site Investigator	Dr. Ingrid Estmann, MD	Universidad Autónoma de Nuevo León, San Nicolás de los Garza, Mexico
LATAM Site Investigator	Elias Fernandez, MD	Universidad de Concepción, Concepción, Chile
Local Site Investigator	Hubert Fernandez, MD	Cleveland Clinic, Cleveland, OH
Local Site Investigator	Jeanne Feuerstein, MD	University of Colorado, Boulder, CO
Genetics Counseling Core	Katherine Fiallos, CGC3	Indiana University, Indianapolis, IN
Core Team	Megan Finke	Parkinson's Foundation, New York, NY
Local Site Investigator	Nicholas Fleming, MD	Atrium Health, Charlotte, NC
Genetics Counseling Core; National Recruiting Site Investigator; Steering Committee	Tatiana Foroud, PhD,	Indiana University, Indianapolis, IN
Genetics Testing Core	Harry Gao, PhD	Fulgent Genetics, Temple City, CA
Core Team Lead; Steering Committee	Kamalini Ghosh Galvelis, MS	Parkinson's Foundation, New York, NY
Local Site Investigator	Ro'ee Gilron, PhD	Rune Labs, San Francisco, CA
Local Site Investigator	Steven Gunzler, MD	Case Western, Cleveland, OH

PDGENE Affiliation	Name	Affiliation(s)
Past Steering Committee	Anne Hall, MD	Parkinson’s Foundation, New York, NY
Local Site Investigator	Deborah Hall, MD	Rush University, Chicago, IL
Local Site Investigator	Ihtsham Haq, MD	University of Miami, Miami, FL
Local Site Investigator	Sharon Hassin, MD	Chaim Sheba Medical Center, Ramat Gan, Israel
Genetics Counseling Core	Laura Heathers	Indiana University, Indianapolis, IN
Local Site Investigator	Emily Hill, MD	University of Cincinnati, Cincinnati, OH
Genetics Counseling Core	Priscila Hodges, MS, CGC3	Indiana University, Indianapolis, IN
Local Site Investigator	Anna Hohler, MD	BMC Community Hospital, Boston, MA
Local Site Investigator	Stuart Isaacson, MD	Parkinson's Disease & Movement Disorders Center of Boca Raton, Boca Raton, FL
LATAM Site Investigator	Dr. Marcelo Kauffman, MD, PhD	Hospital Ramos Mejia, Buenos Aires, Argentina
Local Site Investigator	Tarannum Khan, MD	Cleveland Clinic Weston, Weston, FL
Local Site Investigator	Yasaman Kianirad, MD	University of Illinois-Chicago, Chicago, IL
Local Site Investigator	Annie Killoran, MD	University of Iowa, Iowa City, IA
Local Site Investigator	Sushma Kola, MD	Allegheny Health Network, Pittsburg, PA
Site and Clinical Data Management Core; Steering Committee	Sarah Lawrence, MS	Navitas Clinical Research, Rockville, MD
Site and Clinical Data Management Core	Susan Li, MD, PhD	Navitas Clinical Research, Rockville, MD
Local Site Investigator	Tsao-Wei Liang, MD	Thomas Jefferson University, Philadelphia, PA
Local Site Investigator	Irene Litvan, MD	University of California San Diego, La Jolla, CA
Site and Clinical Data Management Core	Yun Lu, PhD	Navitas Clinical Research, Rockville, MD
Past Steering Committee	Karen S. Marder, MD, MPH	Columbia University, New York, NY
Local Site Investigator	Zoltan Mari, MD	Cleveland Clinic Las Vegas, Las Vegas, NV

PDGENE Affiliation	Name	Affiliation(s)
Local Site Investigator	Connie Marras, MD, PhD	Toronto Western Hospital, Toronto, ON
Steering Committee	Ignacio Mata, PhD	Cleveland Clinic, Cleveland, OH
Local Site Investigator	Kathleen McKee, MD	Intermountain Healthcare, Salt Lake City, UT
National Recruiting Site Investigator; Local Site Investigator; Steering Committee	Niccolo Mencacci, MD, PhD	Northwestern University, Chicago, IL
Genetics Testing Core	Yan Meng, PhD	Fulgent Genetics, Temple City, CA
Genetics Counseling Core	Amanda Miller, MS, CGC3	Indiana University, Indianapolis, IN
Local Site Investigator	Kelly Mills, MD	Johns Hopkins University, Baltimore, MD
Local Site Investigator	Janis Miyasaki, MD	University of Alberta, Edmonton, AB
Previous Core Team Lead	Anna Naito, PhD	Parkinson’s Foundation, New York, NY
Local Site Investigator; Steering Committee	Martha Nance, MD	Park Nicollet Struthers Parkinson's Center, Minneapolis, MN
Genetics Counseling Core	Alia Neibaur	Indiana University, Indianapolis, IN
Core Team	Melissa Nicewaner	Parkinson’s Foundation, New York, NY
National Recruiting Site Investigator	Eleni Okeanis Vaou, MD	University of Texas San Antonio (UTSA), San Antonio, TX
LATAM Site Investigator	Jorge Luis Orozco Vélez, MD	Fundación Valle del Lili, Cali, Colombia
Core Team	Sarah Osborne	Parkinson’s Foundation, New York, NY
Local Site Investigator	Jill Ostrem, MD	University of California San Francisco, San Francisco, CA
Local Site Investigator	Rajesh Pahwa, MD	University of Kansas, Lawrence, KS
Local Site Investigator	Gian Pal, MD	Rutgers University, New Brunswick, NJ
Local Site Investigator; National Recruiting Site Investigator	Ariane Park, MD	Ohio State University Medical Center, Columbus, OH
LATAM Site Investigator	Dr. Susana Lissette Peña Martinez, MD	Universidad Dr. Andrés Bello, San Salvador, El Salvador

PDGENE Affiliation	Name	Affiliation(s)
Steering Committee	John Poma	Parkinson’s Foundation, New York, NY
Local Site Investigator	Joseph Quinn, MD	Oregon Health & Science University, Portland, OR
Site and Clinical Data Management Core	Uma Ragunathan, MS	Navitas Clinical Research, Rockville, MD
Local Site Investigator	Gonzalo Revuelta, DO	Medical University of South Carolina, Charleston, SC
Local Site Investigator	Giulietta Riboldi, MD	New York University, New York, NY
Local Site Investigator	Bernardo Rodrigues, MD PhD	University of Connecticut, Mansfield, CT
LATAM Site Investigator	Dr. Mayela Rodríguez Violante, MD	Instituto Nacional de Neurología y Neurocirugía, Ciudad de México, México
Core Team	Joshua Ruffner	Parkinson’s Foundation, New York, NY
Genetics Counseling Core	Malia Rumbaugh	Indiana University, Indianapolis, IN
LATAM Site Investigator	D. Paula Saffie Awad, MD, PhD	Clínica Santa María, Santiago, Chile
Steering Committee	Ruth Schneider, MD	University of Rochester, Rochester, NY
Local Site Investigator	Julie Schwartzbard, MD	Aventura Neurologists, Aventura, FL
Steering Committee	Michael Schwarzschild, MD, PhD	Massachusetts General Hospital, Boston, MA
Local Site Investigator	Holly Shill, MD	Barrow Neurological Institute, Phoenix, AZ
Local Site Investigator	Lisa Shulman, MD	University of Maryland, College Park, MD
Local Site Investigator	David Simon, MD, PhD	Beth Israel Deaconess Medical Center, Boston, MA
Past Steering Committee	Tanya Simuni, MD	Northwestern University, Chicago, IL
Local Site Investigator	Claudia Testa, MD, PhD	University of North Carolina - Chapel Hill, Chapell Hill, NC
Core Team	Max Thom	Parkinson’s Foundation, New York, NY
Genetics Counseling Core	Michelle Totten	Indiana University, Indianapolis, IN
Genetics Testing Core	Erica Trinh	Fulgent Genetics, Temple City, CA

PDGENE Affiliation	Name	Affiliation(s)
Local Site Investigator	Blanca Valdovinos, MD	University of Rochester, Rochester, NY
Local Site Investigator	Nora Vanegas, MD	Baylor College of Medicine, Houston, TX
Local Site Investigator	Okeanis Vaou, MD	University of Texas San Antonio, San Antonio, TX
Genetics Counseling Core	Jennifer Verbrugge, MS, CGC3	Indiana University, Indianapolis, IN
Local Site Investigator	Rebecca Williamson, MD, PhD	University of Pennsylvania, Philadelphia, PA
Local Site Investigator; Past Steering Committee	Anne-Marie Wills, MD	Massachusetts General Hospital, Boston, MA
Local Site Investigator	Katherine Wong, MD	University of Southern California, Los Angeles, CA
Local Site Investigator	Tao Xie, MD, PhD	University of Chicago, Chigaco, IL
Local Site Investigator	Gilad Yahalom, MD	Shaare Zedek Medical Center, Jerusalem, Israel
Core Team	Addison Yake	Parkinson’s Foundation, New York, NY
Local Site Investigator	Tritia Yamasaki, MD, PhD	University of Kentucky, Lexington, KY
Local Site Investigator	Hengameh Zahed, MD, PhD	Stanford University, Stanford, CA
Local Site Investigator	Elizabeth Zauber, MD	Indiana University, Indianapolis, IN

Alphabetical order by last name.
